# Endovascular exclusion of ascending aortic pseudoaneurysm with an experimental ascending aortic stent-graft

**DOI:** 10.1016/j.xjtc.2023.03.023

**Published:** 2023-04-18

**Authors:** Chibueze Onyemkpa, Joseph S. Coselli

**Affiliations:** a Division of Cardiothoracic Surgery, Michael E. DeBakey Department of Surgery, Baylor College of Medicine, Houston, Tex; b The Texas Heart Institution, Houston, Tex; c CHI St Luke's Health–Baylor St Luke's Medical Center, Houston, Tex; d Cardiovascular Research Institute, Baylor College of Medicine, Houston, Tex

**Figure:**
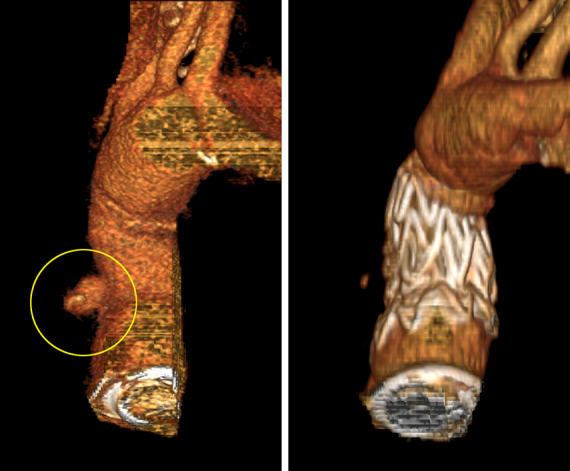
Reconstructed computed tomography image shows the pseudoaneurysm and its exclusion.

**Central Message**

A 60-year-old man with chronic DeBakey type I aortic dissection developed a pseudoaneurysm of the composite valve graft after an aortic root replacement. He underwent exclusion of the aneurysm.

Pseudoaneurysms are uncommon, life-threatening complications that can develop after aortic or cardiac surgery, trauma, or infection. They represent a chronic aortic leak contained by thrombus and fibrosis; urgent surgical repair is needed due to a high risk for frank rupture. Historically, pseudoaneurysms are treated with open repair.^1^ However, contemporary aortic repair may utilize open or endovascular approaches to tailor the intervention to the patient. We present the novel use of an experimental, ascending aortic stent-graft for endovascular exclusion of a proximal ascending aortic pseudoaneurysm, and the later removal of this device as part of a subsequent repair to treat progressive aortic disease related to chronic dissection. This report was prepared in accordance with a clinical research protocol approved by Baylor College of Medicine Institutional Review Board (BCM H-18095; approved February 21, 2006). Written informed consent for publication of study data was obtained from the patient.

## Case Description

**Figure 1:**
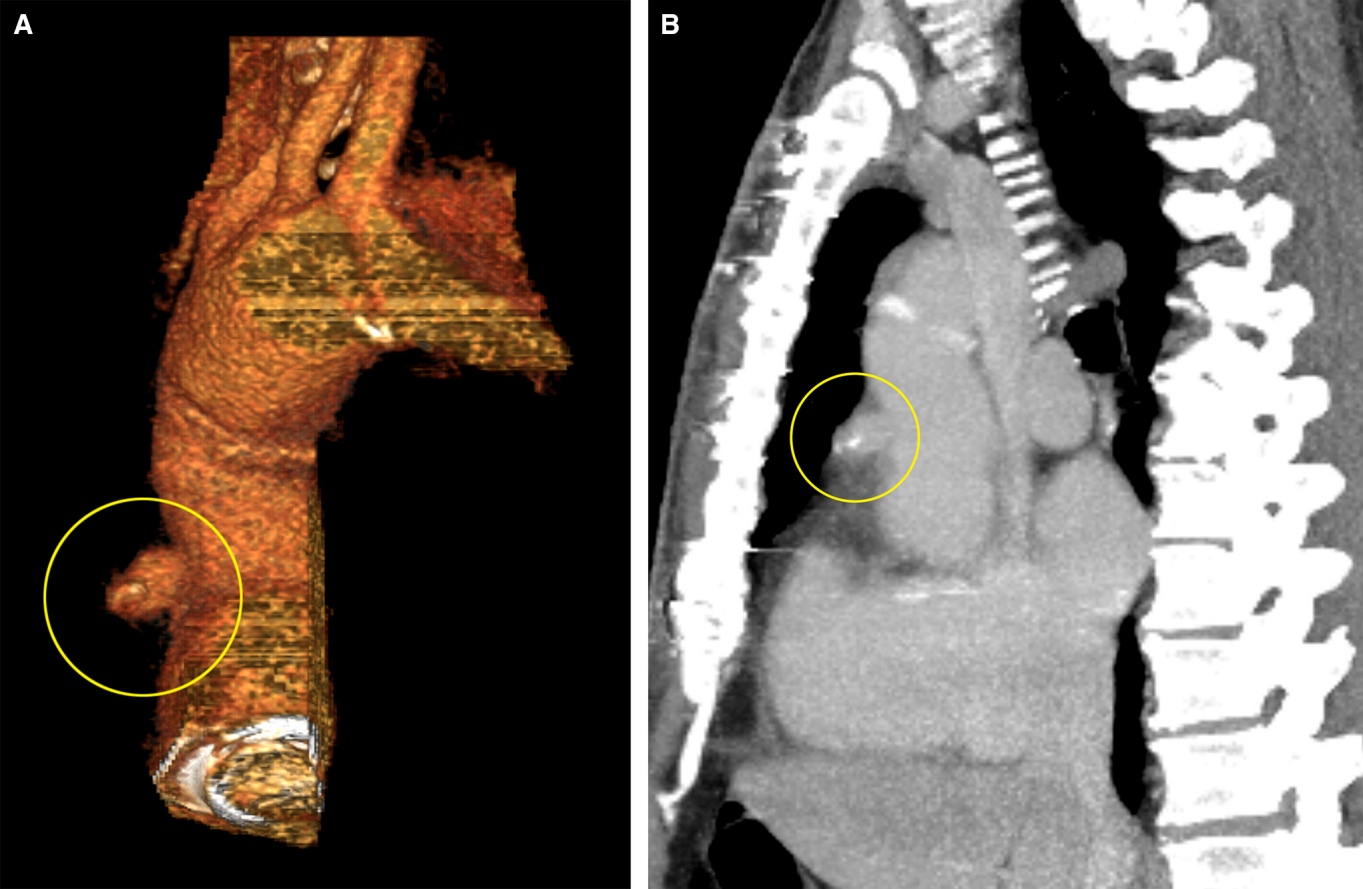
Computed tomography image showing the pseudoaneurysm (*encircled in yellow*) in a 3-dimensionally reconstructed (A) and sagittal (B) view.

The patient was a 60-year-old man with a history of hypertension, class I obesity, and chronic DeBakey type I aortic dissection. At an outside institution, he underwent aortic root replacement using a composite valve graft (CVG) comprising a 29-mm mechanical valve and Valsalva graft. He presented to us 6 months later with a pseudoaneurysm on the anterior aspect of the CVG due to a defect in the graft shown in Figure 1. Considering the likelihood for subsequent open repair to treat the residually dissected transverse aortic arch, we opted for an endovascular repair to address the pseudoaneurysm.

**Figure 2:**
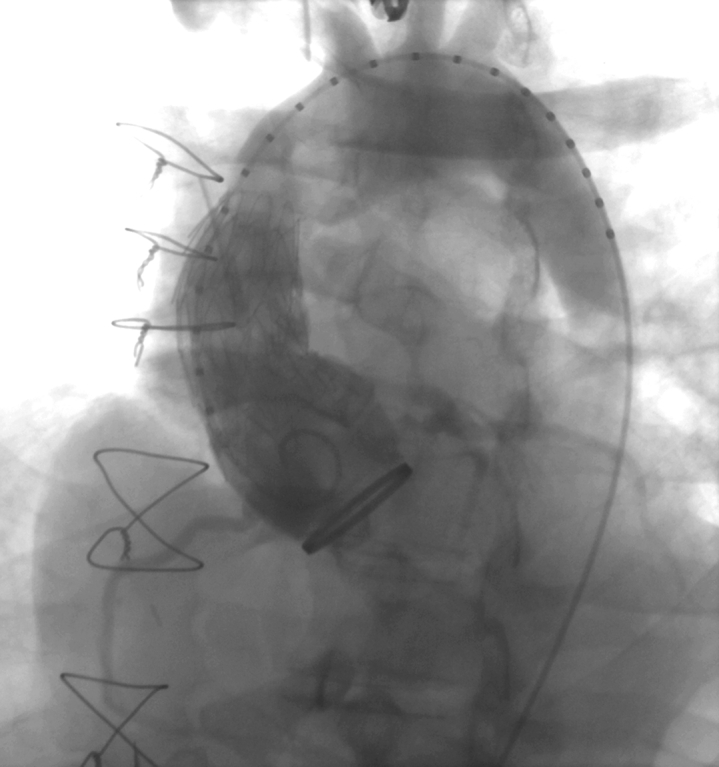
Fluoroscopy image immediately after deployment of experimental ascending aortic stent-graft.

This approach temporarily avoids a redo sternotomy and bridges the patient to a future definitive repair; hence, arguably decreasing the morbidity of future interventions by reducing the number of redo sternotomies. Based on compassionate grounds, we proposed the use of a novel endovascular prosthesis. This experimental 40-mm × 7-cm endovascular ascending aortic stent-graft was deployed to exclude the pseudoaneurysm, successfully addressing this late complication of prior open repair. Access was obtained percutaneously using ultrasound guidance and a 6Fr left common femoral artery (CFA) sheath was inserted. The right CFA was exposed by groin cutdown for device deployment. A 6Fr sheath was then inserted into the right CFA, and an angled guidewire with a Bern catheter was advanced into the ascending aorta. A pigtail catheter was then advanced via the left CFA. An angiogram of the ascending aorta and transverse aortic arch was used to delineate the level of the aortic valve, the left main coronary artery, and the precise location of the pseudoaneurysm. The right CFA was dilated with multiple dilators over a stiff wire and a 24Fr sheath placed. The experimental endograft designed for the ascending aorta (Arise; WL Gore & Associates) was advanced through the 25Fr sheath and deployed in the usual fashion. The device was deployed 3.5 mm and 3.6 mm distal to the right and left main coronary ostia, respectively, and proximal to the origin of the innominate artery; the stent-graft was deployed entirely within the synthetic graft of the original CVG. In this manner, the pseudoaneurysm was completely covered without compromising the coronary arteries or the innominate artery (Figure 2). No ballooning was necessary and completion angiography showed a well-positioned device. Details of our technique have been described previously.^2^ Following satisfactory exclusion, the patient was closely monitored by adhering to an imaging surveillance protocol, with the expectation that additional aortic repair would likely be needed in the future because of chronic dissection in the residual native aorta.

Two years later, he presented with a 6.5-cm arch aneurysm. This progressive expansion of the aortic arch and the proximal portion of the descending thoracic aorta were treated using a modified frozen elephant trunk approach that combined open and endovascular approaches. First, we performed a total arch replacement using a 26-mm collared graft with an elephant trunk graft extension into the descending thoracic aorta. This soft trunk placed in the true lumen was expanded by deploying a 31-mm × 10-cm stent-graft over a guidewire in an antegrade fashion under direct vision. The left subclavian artery was bypassed, and the collared graft used for the distal anastomosis proximal to the left subclavian artery. The left common carotid and innominate arteries were reimplanted using an island technique. The previous ascending aortic stent-graft was removed and a graft-to-graft proximal anastomosis between the previous CVG and the new replacement graft completed. The prosthetic valve remained intact within the previously replaced aortic root. The patient was discharged home without complications on postoperative day 12 and has done well in the 18 months since his aortic repair. He remains under surveillance.

## Discussion

Aortic pseudoaneurysms are associated with a high rate of morbidity and mortality largely driven by the high risk of rupture. These largely arise from suture lines as a result of poor surgical technique or infections. Therefore, proper handling of the aorta, suture technique that avoids unnecessary tension, and asepsis are crucial in preventing the occurrence of pseudoaneurysms. Traditionally, open surgical management is the primary management modality^1^; however, high-risk patients may require other management modalities with more acceptable risks.^2^, ^3^, ^4^, ^5^

Endovascular management of ascending aorta pathologies have become an area of interest because it leverages the typically lower risk of an endovascular approach.^3^, ^4^, ^5^ In the index case, the patient already had a sternotomy and was anticipated to—and did have—a repeat sternotomy to tackle his enlarging aortic arch aneurysm. Subjecting him to 2 prior sternotomies before the arch reconstruction would have significantly increased operative risk. Unfortunately, ascending aortic endografts are not yet readily unavailable; hence, surgeons have been limited to the off-label use of descending thoracic and abdominal aortic stent-grafts.^2^^,^^3^ In this case, we appealed for the use of an experimental ascending aortic endograft currently being used in the Gore Arise trial and the device was approved for use on compassionate basis.

Among the benefits of using the selected endograft in this patient is that it obviates the need to modify the device before use as it is specifically designed for the ascending aorta. This is unlike the off-label use of stent-grafts designed for the descending thoracic or abdominal aorta that require alterations for optimal outcome.^2^ In most cases, length adjustments of the endografts are required; however, other challenges of the ascending aorta such as its curvature and proximity to various crucial structures contribute to the difficulty.^5^ The experimental device used in this case has an innate curvature structured to match the ascending aorta, and the endograft can be partially deployed and modified in situ to allow proper alignment with the curve of the aorta. So far, the use of ascending aorta stent-grafts has shown promising results.^2^^,^^4^^,^^5^

The presence of a mechanical valve CVG in the index repair facilitated the decision use this experimental stent-graft. We were able to get extremely close to the reattached coronary arteries because of the relatively low profile of the valve. If the CVG had a stented bioprosthetic valve instead, it would have made this approach more difficult to adopt; very likely, the expanded profile (ie, the larger framework) of a stented bioprosthetic valve would have made it difficult to fully land the experimental device within the existing synthetic graft.

In addition to the use of descending thoracic and abdominal aortic endografts, other endovascular approaches to ascending aortic pseudoaneurysms that have been attempted include use of the Amplatzer occluder device.^2^^,^^3^ Documented in case reports and some case series, results on its use have been mixed.^2^^,^^3^ In our experience, failure to achieve occlusion required the use of a stent to exclude the pseudoaneurysm.^2^ It is worth noting that off-label use of descending thoracic and abdominal stent-grafts face the risk of failure due to the anatomic configuration of the ascending aorta—especially the enhanced risk of endoleak. Results of the Arise trial will furnish details on the frequency of such failure with stent-grafts specifically designed for the ascending aorta.

## Conclusions

Pseudoaneurysms in the ascending aorta, although infrequent, pose a major challenge to aortic surgeons and demand surgical repair. In patients who are deemed inoperable or at high risk for surgery, or when a future redo sternotomy is anticipated, repair with an endovascular stent-graft is a generally safer approach and can reduce the number of open reoperations. The development of stent-grafts designed specifically for the ascending aorta will likely reduce complications associated with off-label use of other endografts.

Approximately 21 months after our patient underwent total aortic arch replacement with a frozen elephant trunk extension into the proximal portion of the descending thoracic aorta, he underwent a Crawford extent II thoracoabdominal aortic repair. Notable features of the repair included incorporating the stent-graft from the prior frozen elephant trunk into the proximal suture line. With this repair, the patient has had his entire aorta replaced—from the aortic root to the aortic bifurcation. At a 6-month follow-up to his most recent repair, the patient was doing remarkably well and considered himself “100% normal,” having resumed normal activities and avoiding extreme physical exertion.
